# Do Specialized Medical LLMs Demand a Radically New Approach Under the EU’s Medical Device Regulation?

**DOI:** 10.1017/jme.2025.10136

**Published:** 2025

**Authors:** Hannah Louise Smith, W. Nicholson Price

**Affiliations:** 1 Centre for Advanced Studies in Bioscience Innovation Law, Faculty of Law, University of Copenhagen, Denmark; 2 Faculty of Law, University of Michigan, United States

**Keywords:** Large Language Models, Risk Management, Artificial Intelligence, Medical Device Regulation, AI governance, Practice of Medicine

## Abstract

We examine the arguments made by Onitiu and colleagues concerning the need to adopt a “backward-walking logic” to manage the risks arising from the use of Large Language Models (LLMs) adapted for a medical purpose. We examine what lessons can be learned from existing multi-use technologies and applied to specialized LLMs, notwithstanding their novelty, and explore the appropriate respective roles of device providers and regulators within the ecosystem of technological oversight.

Artificial intelligence (AI) is already changing medicine rapidly, but generative AI, including large language models (LLMs), is propelling these developments at a truly breakneck pace. One challenge is how to ensure these developments produce benefits for patient care and medical practice, rather than exposing them to unacceptable risks. Regulators are grappling with the conundrum of how to achieve this in the context of AI, including specialized medical LLMs. The US has done little legislatively, and the Food and Drug Administration has so far favored a cautious approach over wholesale reform. The EU’s legislative efforts, by contrast, have seemingly attempted to match the rapid pace of AI-related innovation, most notably with the introduction of the AI Act^1^ and the updates to the Medical Devices Regulation (MDR)^2^. How well do these frameworks work for specialized medical LLMs?

Not particularly well, argue Onitiu, Wachter, and Mittelstadt in “Walking Backward to Ensure Risk Management of Large Language Models in Medicine.”^3^ They highlight deficiencies in current approaches to risk identification and mitigation to propose an intriguingly broader, more flexible approach to regulators. Two related questions spring from this analysis. First, what can existing multi-use technologies teach us about regulating specialized LLMs, despite their novelty? Second, how much weight should rest with device providers and regulators within the ecosystem of technological oversight?

## Why Walk Backward?

Onitiu and colleagues describe the EU MDR’s current risk management approach as “forward-walking,” in which providers (in EU parlance, those who make a medical device available) follow a sequential order to identify a device’s intended use, mitigate any associated risks, and monitor its safety and performance. But specialized medical LLMs, they find, have general capabilities that are incompatible with this approach. The authors identify three tensions between the forward-walking approach to risk management and specialized medical LLMs. First, the general-purpose origins of these LLMs mean certain user prompts might introduce new purposes that were not assessed by the current approach. Second, resisting a dynamic approach to defining intended uses could, from the outset, lead to errors in risk estimation and mitigation. The authors argue that focusing on a fixed intended use overlooks how the specialized LLM’s capabilities introduce a “variability of risks across a broad spectrum,” which undermines the effectiveness of risk control and evaluation under a forward-walking approach. Third and finally, the adaptive nature of specialized medical LLMs, even with little to no human intervention to prompt such adaptations, challenges providers’ abilities to monitor the device’s safety and effectiveness.

Accordingly, the authors propose an alternative “backward-walking” approach to risk management that responds to the dynamic nature of specialized LLMs. This starts by acknowledging the potential for specialized medical LLM interactions to generate further uses beyond the provider’s intended uses. The authors thus advocate for providers to focus on a broader range of “reasonably foreseeable” uses, rather than intended uses. This leads to an expansive stage of model evaluation and risk mitigation based on the “actual” tasks that are “reasonably foreseeable” for the model to undertake. A backward-walking approach also requires producers to consider the model’s ability to evolve rapidly, even unpredictably, by implementing further real-time monitoring and clearer guidance on how to predetermine model updates whilst still respecting the device’s intended use.

## Existing Multi-Use Technologies and the Regulatory Ecosystem

Onitiu and colleagues make a timely contribution to the literature concerning regulatory challenges posed by novel and emerging technologies in healthcare. Indeed, the authors’ call for an alternative approach to risk management is grounded in the novelty of LLMs and AI, which partly supports its promise to revolutionize healthcare and the practice of medicine. This is a familiar move in regulatory, policy, and academic literature. And of course, it invites a familiar reply: how new is AI, really? A focus on novelty risks obscuring similarities between specialized medical LLMs and existing medical devices that can, and should, help inform any regulatory response.

For example, discussions about potential uses beyond what the developer defines as the device’s intended uses implies this is a unique situation for specialized medical LLMs. However, differences between potential capabilities and intended uses exist in other medical devices — differences accepted (if debated) within the current risk management approach. This is not uncharted territory; the repurposing of niche-busters^4^ and off-label usage of other devices or medicines^5^ provide fruitful avenues for learning from real-world experiences to draw parallels. Across a range of medical technologies, regulators and practitioners already must grapple with the challenge of clinicians and patients who use the technologies outside their “intended uses.” It is challenging to draw the contestable line between what limits regulators may and should impose on the creators of medical technologies versus what questions remain firmly embedded within the practice of medicine, and therefore subject to a different set of constraints,^6^ and that challenge predates the introduction of AI into medicine. Other more closely related technological developments, such as connected intelligent medical devices or other examples of AI as a medical device, may have a changing risk profile throughout their lifecycle that can also provide insights for regulating specialized medical LLMs.^7^

To be sure, specialized medical LLMs may still present some novel challenges, mainly through the greater breadth of their potential capabilities and an increased blurriness between “technical capabilities” and “intended uses” in which LLMs engage in open-ended dialogues. In particular, some aspects of control simply may not fit well within a principally *ex ante* regulatory approach. Given the flexibility of an LLM, it seems remarkably difficult to distinguish in a principled way between “reasonably foreseeable” uses and “actual uses” in the real world of growing datasets, shifting practices, and differences among clinicians and environments. Trying to account for so many possibilities, mostly *ex ante*, risks tipping the regulatory approach from dynamic to unworkable. At the very least, identifying emergent risks will entail substantial monitoring of LLM use, effectiveness, and problematic outcomes. Sometimes, this may be within the capabilities of system creators or providers — but often understanding and shaping the final implementation steps will instead fall to health systems.^8^ Specialized medical LLMs (and other medical AI) may indeed demand a new look at systems to ensure their safety and effectiveness in improving patient health, but that doesn’t necessarily mean that all the altered oversight should be localized within device agencies, or that dispositive weight should be placed on forecasting how users may end up employing these powerful tools.

## Conclusion

There is a need for further interdisciplinary conversations to better determine the aspects of specialized medical LLMs that are truly novel and how these novel aspects impact the current governance framework. It is particularly pertinent in this context because the assessment fits in a broader framework that also includes the EU’s AI Act^9^ and Product Liability Directive.^10^ This interconnected regulatory framework also draws attention to the plethora of actors in the space. Efforts to alter the risk management of medical devices must explicitly explore how appropriate it is for the providers to have further power over the regulation of medical devices, a development that logically stems from the heightened responsibilities that a backward-walking risk management approach would necessitate. Relying on providers to resolve these issues alone may ultimately be ineffective, increasing risks to patient health, and decreasing the odds of positive transformation in the health system.
